# Construction of an Artificial Interfacial Layer with Porous Structure toward Stable Zinc‐Metal Anodes

**DOI:** 10.1002/smsc.202300007

**Published:** 2023-04-12

**Authors:** Xianhong Chen, Xiaodong Shi, Pengchao Ruan, Yan Tang, Yanyan Sun, Wai-Yeung Wong, Bingan Lu, Jiang Zhou

**Affiliations:** ^1^ School of Materials Science and Engineering Hunan Provincial Key Laboratory of Electronic Packaging and Advanced Functional Materials Central South University Changsha Hunan 410083 China; ^2^ China State Key Laboratory of Marine Resource Utilization in South China Sea Hainan University Haikou Hainan 570228 China; ^3^ Department of Applied Biology & Chemical Technology and Research Institute for Smart Energy The Hong Kong Polytechnic University Hong Kong China; ^4^ School of Physics and Electronics Hunan University Changsha Hunan 410082 China

**Keywords:** NH_4_V_4_O_10_, organic acid etching, surface modification, zinc-metal anodes, zinc-ion batteries

## Abstract

Aqueous zinc‐ion batteries possess great potential in stationary energy storage devices. Nevertheless, the occurrence of zinc dendrite growth and hydrogen evolution reaction severely hinders the utilization efficiency and service life of zinc‐metal anodes. Herein, an in situ etching strategy is proposed to construct an interfacial layer with porous structure on the surface of zinc foil under the assistance of tartaric acid (denoted as TA@Zn). The optimized anode surface is beneficial to not only achieve uniform Zn deposition behavior due to the low nucleation overpotential, but also enhance the interfacial reaction kinetics due to the reduced activation energy barrier. As expected, the TA@Zn‐based symmetric cell delivers small voltage hysteresis and superior stability for 5000 h at the current density of 1 mA cm^−2^. Moreover, the TA@Zn|NH_4_V_4_O_10_ cell also exhibits high specific capacity and long‐term cycling stability.

## Graphical abstract

Artificial interfacial layers with porous structures are constructed on the surface of Zn foil via an etching treatment with tartaric acid and they endow TA@Zn with low nucleation overpotential and low activation energy, thereby enabling uniform Zn^2+^ deposition behavior and enhancing interfacial reaction kinetics.

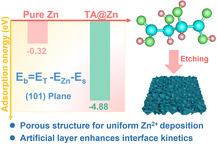

## 1 Introduction

Aqueous zinc‐ion batteries (ZIBs) have shown great potential in the domain of large‐scale energy storage due to the high safety and low cost, resulting from the use of nonflammable and nontoxic electrolyte.^[^ ^1^ ^]^ In view of industrial applications, metallic zinc is an ideal anode material for ZIBs due to the advantages of low potential, high gravimetric capacity, and abundant reserves.^[^ ^2^ ^]^ Nevertheless, the practical applications of zinc‐metal anodes are still hindered by some intractable issues, such as dendrite growth and hydrogen evolution reaction, thereby leading to low utilization efficiency and unsatisfactory performance.^[^ ^3^ ^]^

Currently, extensive attention has been concentrated on regulating the interfacial reactions between electrolyte and zinc anode, including concentration and composition optimization of electrolyte,^[^ ^4^ ^]^ as well as construction of artificial interfacial layer.^[^ ^5^ ^]^ The construction of artificial interfacial layer on the surface of zinc metal is an economical and practical strategy to promote reversibility and durability. However, the artificial interfacial layers are normally constructed through ex situ methods, such as knife coating,^[^ ^6^ ^]^ evaporation coating,^[^ ^7^ ^]^ sputter coating,^[^ ^8^ ^]^ spin coating,^[^ ^9^ ^]^ and chemical vapor deposition,^[^ ^10^ ^]^ which always suffer from the issues of uneven coating, increased electrode thickness, weak adhesion, and easy failure. These issues may result in irregular deposition behavior of Zn^2+^, low energy density, and short cycle life of batteries.^[^ ^11^ ^]^ In this regard, in situ formation of artificial interfacial films (e.g., ZnO,^[^ ^12^ ^]^ ZnS,^[^ ^13^ ^]^ ZnSe,^[^ ^14^ ^]^ ZnP^[^ ^15^ ^]^) has been proposed to address the above problems to some extent, whereas the complex preparation process and low preparation efficiency still limit their practical applications. Therefore, it is necessary to develop a facile and effective strategy for manipulating the surface properties of zinc anode, eliminating the effects of side reactions and guaranteeing reversible plating/stripping behavior.^[^ ^16^ ^]^

Tartaric acid is one of the major organic acids in grape wine, and the pH value of its saturated solution is about 3.56 at 25 °C, which can react with zinc metal through chemical replacement reaction and generate artificial interfacial film rich in zinc tartrate (C_4_H_4_O_6_Zn). The surface of zinc metal can transform from dense structure to porous structure after acid treatment, which is beneficial for uniform electric field distribution, low nucleation barrier, and fast ionic migration.

Herein, an artificial interfacial film with porous structure is introduced on a zinc‐metal anode by immersing commercial zinc foil into tartaric acid solution (denoted as TA@Zn). The in situ protective layer contributes to the fast diffusion of Zn^2+^ and considerable reaction efficiency, while the porous surface conduces to uniform deposition behavior of Zn^2+^. As a result, the TA@Zn|TA@Zn symmetric cells display low potential polarization and ultrastable cycling performance over 5000 and 1000 h at 1 and 5 mA cm^−2^, respectively. Moreover, its practicality is further verified by assembling TA@Zn|NH_4_V_4_O_10_ cells, delivering high reversible capacity of 291.1 and 257.6 mAh g^−1^ at 5 and 10 A g^−1^, respectively. This work may provide new references for the construction of artificial interfacial layer with other metal anodes.

## 2 Results and Discussion

**Figure 1:**
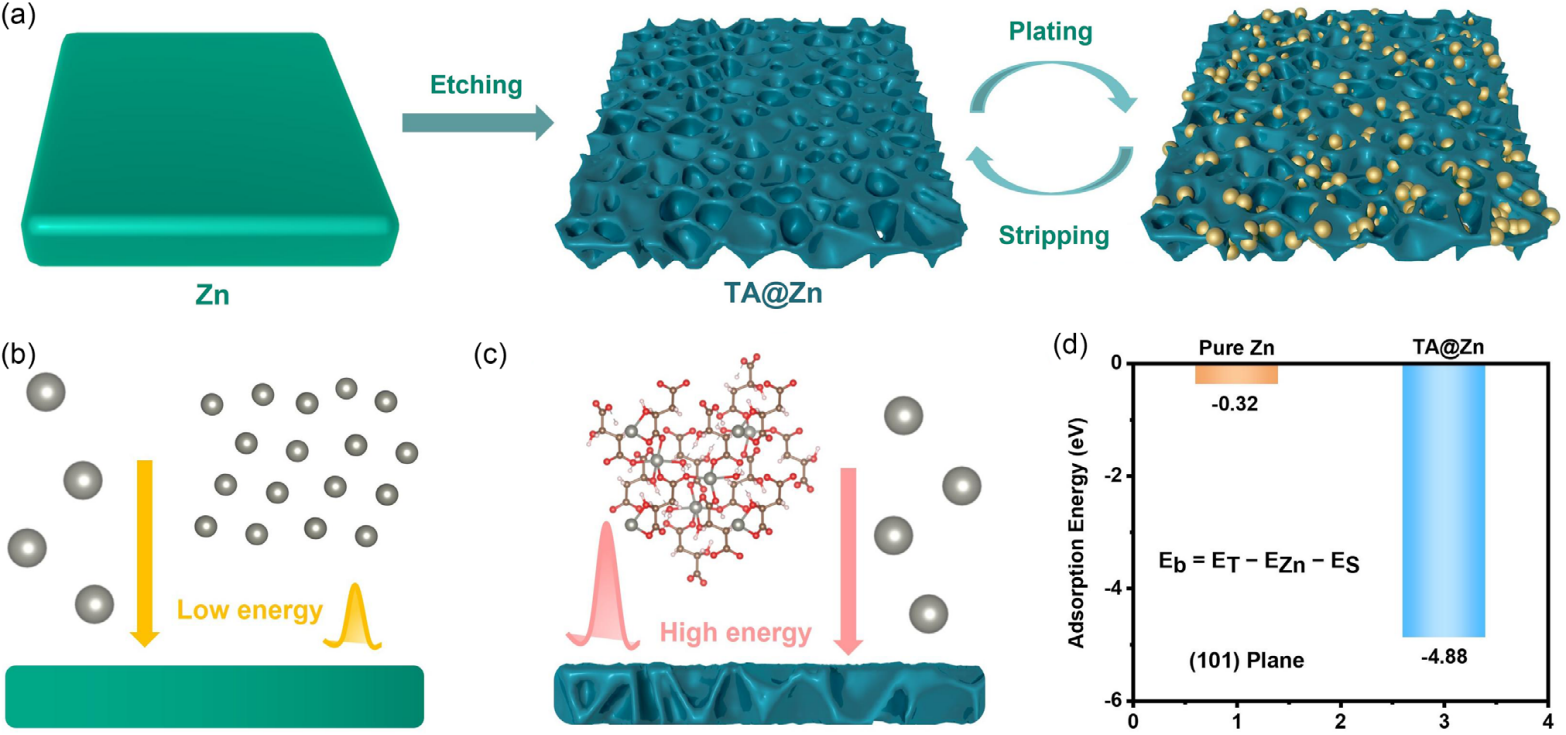
a) Schematic illustration of the tartaric‐acid‐etching zinc‐metal anode. d) Calculated adsorption energy of: b) Zn foil and c) TA@Zn on the (101) plane.

**Figure 2:**
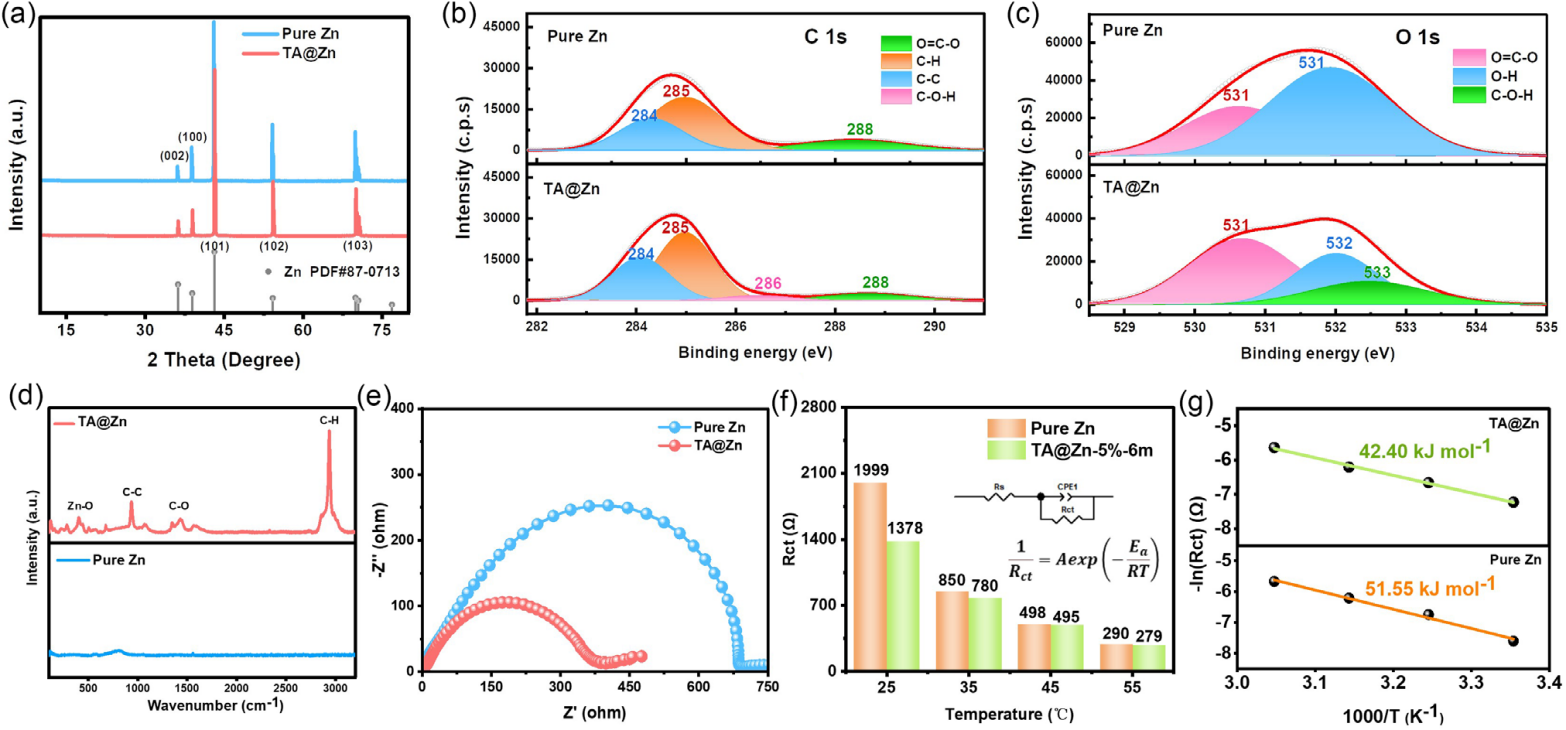
a) XRD patterns of Zn foil and TA@Zn. b,c) High‐resolution C 1s (b) and O 1s (c) spectra of pure Zn and TA@Zn. d) Raman spectra of pure Zn and TA@Zn. e) Electrochemical impedances of pure Zn and TA@Zn. f) Charge transfer resistances of pure Zn and TA@Zn tested at different temperatures and g) their corresponding activation energy.

TA@Zn is prepared by immersing zinc foil into tartaric acid solution with different concentrations for specific time and then washed multiple times with deionized water to remove the residues. As shown in **Figure** **1**a, tartaric acid etching process induces the formation of artificial interfacial layer on the surface of zinc foil with porous structure. Density functional theory (DFT) calculations are performed to obtain the adsorption energy of Zn^2+^ on the (101) plane of Zn foil and TA@Zn (Figure 1b–d). The adsorption energy of Zn^2+^ in the surface of TA@Zn (−4.88 eV) is much lower than that of Zn foil (−0.32 eV), indicating that TA@Zn is more favorable for the absorption of Zn^2+^. In addition, scanning electron microscopy (SEM) is applied to observe the morphology before and after etching, which demonstrates the surface transformation of zinc foil from 2D to 3D structure (Figure S1, Supporting Information). The corresponding energy‐dispersive spectroscopy (EDS) element mapping results confirm the presence of C, O, and Zn in the resultant TA@Zn (Figure S2, Supporting Information). To give a clear explanation of crystal plane behavior, X‐ray diffraction (XRD) is performed and manifests that the ratio of *I*_(002)_/*I*_(100)_ increases from 0.44 for bare Zn to 0.58 for TA@Zn, which shows increased (002) crystal plane and decreased (100) crystal plane, illustrating more exposed (002) crystal plane after acid etching (**Figure** **2**a and S3, Supporting Information). Notably, the reasonable explanation for the undetected zinc tartrate in the artificial film is that large amounts of the generated zinc tartrate are washed off during the washing process, and the residual zinc tartrate content is too low to be detected by XRD.

Meanwhile, X‐ray photoelectron spectroscopy (XPS) is performed to verify the chemical compositions of zinc surface after organic acid etching process. According to the high‐resolution C 1*s* (Figure 2b) and O 1*s* (Figure 2c) spectra of zinc foil with TA@Zn, the surface of TA@Zn has extra C–O–H bond groups (C 1*s*, 286.46 eV; O 1*s*, 532.48 eV), which can be attributed to the residual zinc tartrate.[^5^, ^17^] The decreased intensity of Zn 2*p* peak indicates the successful etching after TA treatment (Figure S4 and Table S1, Supporting Information). The formation of the corresponding zinc compounds is further verified by Raman spectra (Figure 2d). Compared with pure Zn, TA@Zn shows obvious peaks of C–H bond (2936.4 cm^−1^), C–O bond (1431.7 cm^−1^) and C–C bond (933.1 cm^−1^),^[^ ^18^ ^]^ as well as Zn–O (400.1 cm^−1^), all of which can be attributed to the zinc tartrate in the interfacial film.^[^ ^19^ ^]^ In addition, the hydrophilicity of zinc surface is also evaluated by contact angle measurement, as shown in Figure S5, Supporting Information. The intrinsic wettability of TA@Zn is superior to the pure Zn, where the contact angle of TA@Zn is smaller than that of pure Zn from 0 to 180 s apparently. The smaller contact angle reveals better hydrophilicity.

To investigate the effect of acid etching strategy on reaction kinetics, cyclic voltammetry (CV) measurements of symmetric cells were tested at the scan rate of 10 mV s^−1^.^[^ ^20^ ^]^ As shown in Figure S6, Supporting Information, TA@Zn illustrates a larger electrochemical active area compared to pure Zn. Moreover, electrochemical impedance spectroscopy (EIS) is performed to evaluate the charge transfer performance. The charge transfer resistance (*R*_ct_) of TA@Zn is smaller than that of Zn foil (Figure 2e–f and S7, Supporting Information), suggesting faster charge transfer behavior. Besides, the activation energies of TA@Zn and Zn foil have been calculated to compare the transference and desolvation of Zn^2+^ based on their *R*_ct_ values tested at different temperatures as well as Arrhenius equation.^[^ ^12^ ^]^ As summarized in Figure 2g, the activation energies of pure Zn and TA@Zn are calculated to be 51.55 and 42.4 kJ mol^−1^, manifesting that TA@Zn holds faster Zn^2+^ ions desolvation and transfer kinetics. Consequently, the unique 3D‐structured artificial protective layer endows TA@Zn with lower activation energy barrier, which facilitates fast charge/Zn^2+^ transfer and speedy interfacial reaction kinetics.

**Figure 3:**
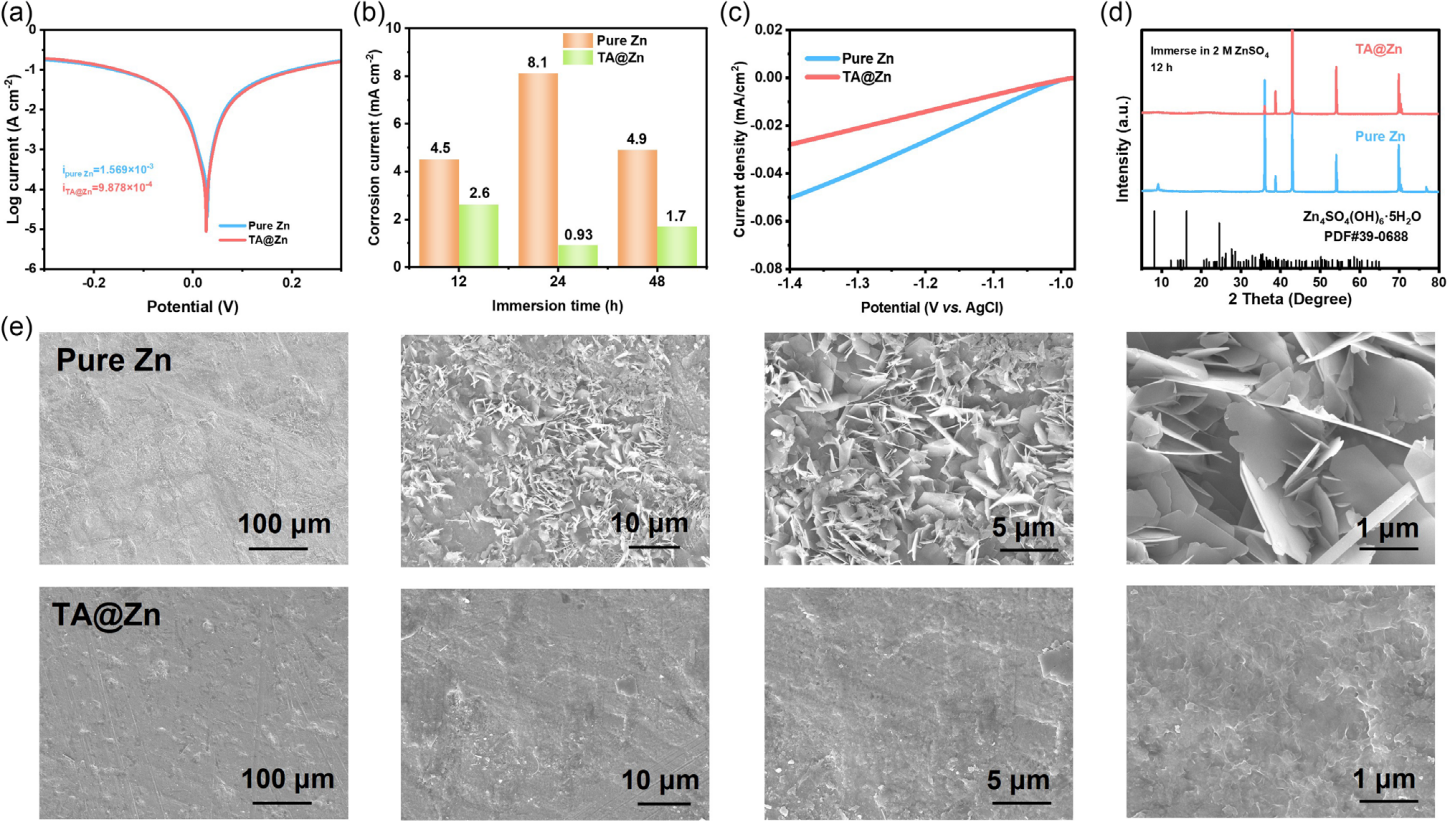
a) Linear polarization curves of the Zn|Zn and TA@Zn|TA@Zn symmetric cells. b) Corrosion current density of pure Zn and the TA@Zn anode after immersing in 2 m ZnSO_4_ electrolyte for 12, 24, and 48 h. c) Linear sweep voltammetry (LSV) profiles of the Zn|Zn and TA@Zn|TA@Zn symmetric cells tested by three‐electrode system. d) XRD patterns of Zn foil and the TA@Zn anode after immersing in 2 m ZnSO_4_ electrolyte for 12 h. e) SEM images of Zn foil and TA@Zn anode after 150 cycles in symmetric cells (current density: 1 mA cm^−2^; areal capacity: 0.25 mAh cm^−2^).

In addition to the reaction kinetics, the interfacial stability of the zinc‐metal anode also plays a main role on ZIBs performance. As shown in **Figure** **3**a and S8, Supporting Information, linear polarization curve is applied to investigate the anticorrosion properties of anode, and TA@Zn shows lower corrosion current density compared with pure Zn (9.878 × 10^−4^ versus 1.569 × 10^−3^ A cm^−2^), implying enhanced corrosion resistance. Additionally, TA@Zn still retains stronger corrosion resistance after immersing TA@Zn in 2 m ZnSO_4_ electrolyte (Figure 3b and S9, Supporting Information). Moreover, the time‐dependent open‐circuit potential curves also reveal the smaller corrosion potential of TA@Zn (Figure S10, Supporting Information). Furthermore, the current density of TA@Zn is smaller in the voltage range than that of pure Zn (Figure 3c). Besides, there is an obvious XRD peak at around 8° belonging to Zn_4_SO_4_(OH)_6_·5H_2_O (ZSH) after immersing Zn foil in 2 m ZnSO_4_ electrolyte for 12 h (Figure 3d), but the peak doesn't appear for TA@Zn. The ZSH by‐products mainly originate from the irreversible side reactions and can lead to low zinc utilization, unstable interface, zinc dendrite growth, and even electrode degradation. In order to intuitively verify the formation of byproducts, the SEM images of Zn foil and TA@Zn anode after 150 cycles in symmetrical cells (current density: 1 mA cm^−2^; areal capacity: 0.25 mAh cm^−2^) have been characterized in Figure 3e. The uneven sheet‐like byproducts can be clearly detected on the surface of Zn foil, while that of TA@Zn still remains relatively smooth. XRD patterns after cycling (Figure S11, Supporting Information) were also evaluated, which correspond to the standard PDF card of ZSH. Besides, the element composition of sheet‐like by‐products is determined by EDS, further confirming the existence of ZSH (Figure S12, Supporting Information).

**Figure 4:**
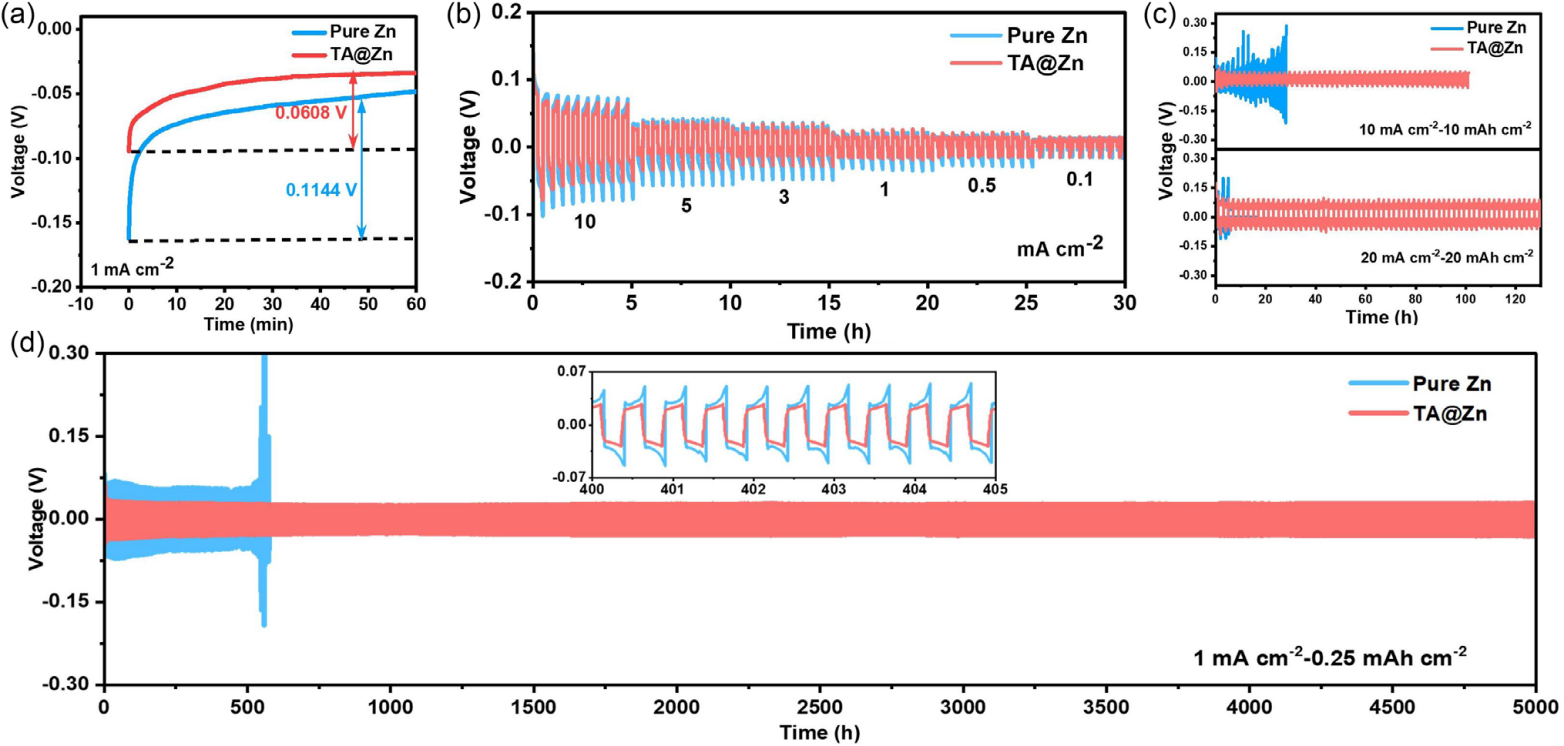
a) Nucleation overpotential of Zn^2+^ at 1 mA cm^−2^ on the surface of pure Zn and TA@Zn. b) Rate performance of Zn|Zn and TA@Zn|TA@Zn symmetric cells. c,d) Cycling performance of Zn|Zn and TA@Zn|TA@Zn symmetric cells at 10 and 20 mA cm^−2^ (c) and 1 mA cm^−2^ (d).

In order to evaluate the diffusion behavior of Zn^2+^ on the surface of the zinc anode, chronoamperometry (CA) curves of symmetric cells were performed. Apparently, the equilibrium deposition current of Zn foil within 1000 s is larger than that of TA@Zn (Figure S13, Supporting Information). The poor stability and large deposition current of pure Zn indicate that Zn^2+^ tends to attach to previously galvanized sites rather than other smooth surfaces. In contrast, TA@Zn has smaller and stable deposition current, which benefits from abundant nucleation sites. Meanwhile, the nucleation overpotential of Zn^2+^ on the surface of TA@Zn is always lower than that of Zn foil (**Figure** **4**a and S14, Supporting Information), with 0.0608 versus 0.1144 V at 1 mA cm^−2^, and 0.0896 versus 0.0914 V at 5 mA cm^−2^. Lower nucleation overpotential can promote the reversible plating/stripping behavior of Zn^2+^ and alleviate the potential polarization during cycling.

The galvanostatic cycling performance of symmetric cells at different test conditions was compared. As depicted in Figure 4d and S15, Supporting Information, the symmetric cell assembled by TA@Zn can achieve ultralong cycling stability for 5000 h with a small voltage hysteresis of 0.03 V, while Zn foil can only last for 500 h with a high voltage hysteresis of 0.07 V at the condition of 1 mA cm^−2^ and 0.25 mAh cm^−2^. Additionally, as expected, the symmetric batteries assembled by the other etched zinc‐metal samples also exhibit small voltage hysteresis and long‐term cycling stability, suggesting the superiority of organic acid‐etching strategy. In addition, the rate performances of symmetric cells at the current density of 10–0.1 mA cm^−2^ have also been measured (Figure 4b), in which TA@Zn delivers smaller voltage hysteresis than Zn foil throughout the cycling process. When the current density and the areal capacity are set as 5 mA cm^−2^ and 1.25 mAh cm^−2^, Zn foil displays continuous voltage fluctuation in the initial 45 h and fails within 100 h, while TA@Zn performs stable voltage hysteresis for 1000 h (Figure S16, Supporting Information). Impressively, TA@Zn also can maintain stable cycling capability for 100 h and 130 h even at 10–10 mAh cm^−2^ and 20–20 mAh cm^−2^ (Figure 4c), verifying the wide operating range and applicability of TA@Zn. Interestingly, TA@Zn‐30%‐6m can maintain stable cycling capability over 80 h at 10–10 mAh cm^−2^, implying that the presence of large holes on the surface of TA@Zn‐30%‐6m brought from adequate etching is beneficial for fast Zn^2+^ plating/stripping (Figure S17, Supporting Information). High corrosion resistance endows TA@Zn with good structural stability at high current density, while low nucleation overpotential conduces to the rapid nucleation and growth process. Additionally, low‐activation energy barrier means fast desolvation process of hydrated Zn^2+^ ions at the electric double layer of TA@Zn anode, thus guaranteeing stable and uniform zinc deposition/stripping behavior in TA@Zn|TA@Zn symmetric cell at high current density. The summary of the galvanostatic performance of pure Zn and optimized zinc metal after different acid treatment is shown in Table S2, Supporting Information.

**Figure 5:**
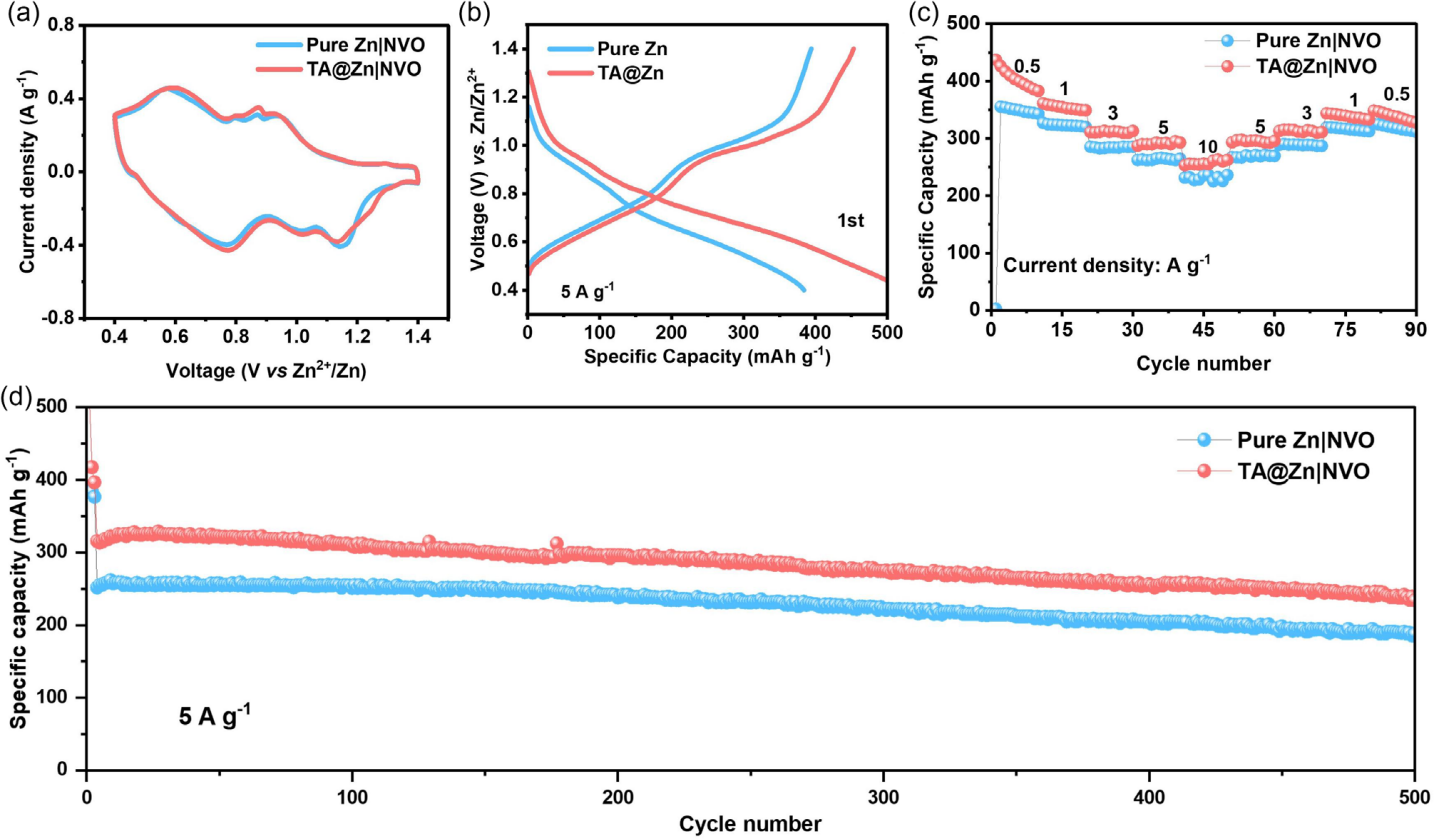
a) CV curves, b) galvanostatic charge and discharge profiles, c) rate performance, and d) cycling performance of Zn|NH_4_V_4_O_10_ and TA@Zn|NH_4_V_4_O_10_ full cells.

In order to further confirm the feasibility and availability of organic‐acid‐etching strategy, NH_4_V_4_O_10_ (NVO) cathode is matched to assemble full cell (Figure S18, Supporting Information). The CV curves of Zn|NVO and TA@Zn|NVO full cells show a typical multistep (de)intercalation reaction of Zn^2+^ (**Figure** **5**a). Figure 5b depicts the galvanostatic charge and discharge profiles at 5 A g^−1^, in which their voltage plateaus correspond to the redox peaks in CV curves. Also, TA@Zn|NVO illustrates the capacity of 404.5, 354.3, 311.4, 291.1, and 257.6 mAh g^−1^ at 0.5, 1, 3, 5, and 10 A g^−1^, respectively, which are obviously higher than those of Zn|NVO full cell (Figure 5c). Furthermore, the cycling performances at 5 A g^−1^ indicate that the zinc storage capacity of TA@Zn|NVO full cell is always higher than Zn|NVO full cell during the 500 cycles (Figure 5d).

## 3 Conclusion

The artificial protective layer with porous structure has been in situ constructed on zinc foil through mild acid‐etching strategy. As a result, the optimized surface of TA@Zn with porous structure enlarges the electrochemical active area that exposes more active sites, thus enhancing the charge/Zn^2+^ transfer behavior and facilitating the reaction kinetics. These positive effects guarantee low activation energy barrier, low nucleation overpotential, as well as uniform zinc deposition behavior. For symmetric cells, TA@Zn anode holds small voltage hysteresis and long‐term stability for 5000 h at 1 mA cm^−2^ and can still maintain stable cycle performance over 130 h even at 20 mA cm^−2^. Importantly, the TA@Zn|NVO cell delivers higher specific capacity and better cycling performance than the cell with commercial zinc foil, further demonstrating the advantages of acid‐etching strategy.

## Conflict of Interest

The authors declare no conflict of interest.

## Supporting information

Supplementary Material

## Data Availability

The data that support the findings of this study are available from the corresponding author upon reasonable request.
