# Construction of an Artificial Interfacial Layer with Porous Structure toward Stable Zinc‐Metal Anodes

**DOI:** 10.1002/smsc.202400100

**Published:** 2024-03-25

**Authors:** Xianhong Chen, Xiaodong Shi, Pengchao Ruan, Yan Tang, Yanyan Sun, Wai‐Yeung Wong, Bingan Lu, Jiang Zhou

*Small Science* **2023**, *6*, 2300007

DOI: 10.1002/smsc.202300007

An error was introduced when the article was prepared for proofing, and a correction was overlooked during the proofing process prior to publication in Early View; the units in the main text in the penultimate paragraph of Section 2 of the published article therefore need to be clarified.

The following sentences are hereby updated:“Impressively, TA@Zn also can maintain stable cycling capability for 100 h and 130 h even at 10–10 mAh cm^−2^ and 20–20 mAh cm^−2^ (Figure 4c), verifying the wide operating range and applicability of TA@Zn. Interestingly, TA@Zn‐30%‐6m can maintain stable cycling capability over 80 h at 10–10 mAh cm^−2^, implying that the presence of large holes on the surface of TA@Zn‐30%‐6m brought from adequate etching is beneficial for fast Zn^2+^ plating/stripping (Figure S17, Supporting Information).”

The revised, clarified text is:

“Impressively, TA@Zn also can maintain stable cycling capability for 100 h at 10 mA cm^−2^ and 10 mAh cm^−2^, and 130 h even at **20 mA cm** ^**−2**^ **and 20 mAh cm** ^**−2**^ (Figure 4c), verifying the wide operating range and applicability of TA@Zn. Interestingly, TA@Zn‐30%‐6m can maintain stable cycling capability over 80 h at **10 mA cm** ^**−2**^ **and 10 mAh cm** ^**−2**^, implying that the presence of large holes on the surface of TA@Zn‐30%‐6m brought from adequate etching is beneficial for fast Zn^2+^ plating/stripping (Figure S17, Supporting Information).”

