# Supplementary material for: Construction of an Artificial Interfacial Layer with Porous Structure toward Stable Zinc‐Metal Anodes
Source: Small Sci. 2023 Apr 12;3(6):2300007. doi: 10.1002/smsc.202300007 (PMC11935934; doi:10.1002/smsc.202300007)
Supplement: Supplementary file 1 — Supplementary Material [file SMSC-3-2300007-s001.pdf]

Supporting Information for

Construction of Artificial Interfacial Layer with Porous Structure toward Superior Zinc Metal Anode

Xianhong Chen, Xiaodong Shi, Pengchao Ruan, Yan Tang, Yanyan Sun,* Wai-Yeung Wong,* Bingan Lu, and Jiang Zhou*

X. Chen, P. Ruan, Y. Tang, Y. Sun, J. Zhou

School of Materials Science and Engineering, Hunan Provincial Key Laboratory of Electronic Packaging and Advanced Functional Materials, Central South University, Changsha, Hunan 410083, China

E-mail: yanyan.sun@csu.edu.cn; zhou_jiang@csu.edu.cn

X. Shi

China State Key Laboratory of Marine Resource Utilization in South China Sea, Hainan University, Haikou, Hainan 570228, China

W-Y. Wong

Department of Applied Biology & Chemical Technology and Research Institute for Smart Energy, The Hong Kong Polytechnic University, Hong Kong, China

E-mail: wai-yeung.wong@polyu.edu.hk

B. Lu

School of Physics and Electronics, Hunan University, Changsha, Hunan 410082, China

1. Experimental section

1.1 Preparation of TA@Zn anode by acid-etching strategy

L-(+)-Tartaric acid (99%) was purchased from Macklin. The molecular formula of tartaric acid is $C_4H_6O_6$. The porous structures and artificial film-modified zinc metal anodes in this work were prepared by immersing commercial zinc foil (thickness: 0.08 mm) in tartaric acid solution. Generally, the higher the concentration of tartaric acid solution, the more acidic it is. Thus, the optimized properties of zinc metal are determined by the concentration of tartaric acid and the immersion time. In this work, 1 g, 2 g, 4 g and 6 g of tartaric acid powder were immersed in 19 mL, 18 mL, 16 mL and 14 mL of deionized water, respectively, *i.e.*, the concentrations of tartaric acid solution were 5 wt.%, 10 wt.%, 20 wt.% and 30 wt.%. By controlling the immersion time (3-6-9 min), a series of surface modified zinc anodes were prepared, systematically named as TA@Zn-concentration-time. For example, TA@Zn-5%-3m represented zinc foil was soaked in 5 wt.% tartaric acid solution for 3 minutes. After the immersion process, the etched zinc foil was washed with deionized water multiple times to clean the tartaric acid residue, and dried in an oven at 60 ^oC for 0.5 h. In this work, the anode sample of TA@Zn-5%-6m presents the best electrochemical performance, and it is marked as TA@Zn throughout the manuscript and supporting information.

1.2 Preparation of $NH_4V_4O_{10}$ cathode

Firstly, 1.17 g of NH_4VO_3 was dissolved into 80^oC deionized water and form the light-yellow solution. Next, 1.891 g of $H_2C_2O_4 \cdot 2H_2O$ powder was slowly added into the yellow solution under the action of a magnetic stirrer, to eventually obtain a black green solution. Then, the solution was transferred to a 50 mL autoclave and kept it at 140^oC for 48h. After the

autoclave was cooled naturally, the solid obtained was removed and rinsed several times with deionized water. Eventually, the product was dried at 60°C for 12 h, then $NH_4V_4O_{10}$ was obtained.

For preparing cathodes, active material $NH_4V_4O_{10}$, Super P and polyvinylidene fluoride (PVDF) binder were mixed in a mass ratio of 7:2:1 and ground at room temperature for 40 minutes. Then, N-methyl-2-pyrrolidone (NMP) solvent was added slowly and milling continued for 20 minutes, to obtain a homogeneous slurry. Finally, the slurry was uniformly coated on stainless steel wire mesh (SSWM) with a diameter of 12 mm, and dried in a vacuum oven at 80°C for 12h. The areal loading density of $NH_4V_4O_{10}$ is about 1.0 mg cm^{-2}.

1.3 Material characterizations

The X-ray diffraction (XRD) patterns of electrodes were performed on X-ray diffractometer (XRD, Rigaku D/max2500). Morphology studied with the corresponding energy dispersive spectrometer (EDS) mapping of electrodes were conducted by scanning electron microscopy (SEM, Quanta FEG 250). X-ray photoelectron spectroscopy (XPS) measurements were employed using a spectrometer (Esca lab 250xi, Thermo Scientific). Raman spectroscopy measurements were performed at room temperature on a spectrometer (Renishaw Micro-Raman Spectroscopy System).

1.4 Electrochemical measurements

Full batteries were fabricated using 2016 type coin cells with Zn metal as the anode, and ammonium vanadates and 2 M $ZnSO_4$ solutions as cathode and electrolyte, respectively. Glass fiber separators (Whatman, GF/D) were used as the membranes. Symmetric batteries were assembled with zinc metal as cathode/anode and 2 M $ZnSO_4$ as electrolyte. The

galvanostatically tests of full batteries were performed on battery testing systems (LAND CT2001A and LANBTS) in the voltage window of 0.4~1.4 V, while that of symmetric batteries were conducted on BTSDAE and LANBTS battery system. The electrochemical performance tests including cyclic voltammetry (CV) tests, electrochemical impedance spectroscopy (EIS), linear polarization curves, linear sweep voltammetry and chronoamperometry (CA) were tested by the CHI 660e and CHI 604e electrochemical station. All the characterizations were performed at room temperature without otherwise specified.

2. Calculation of adsorption energy

The first principle DFT calculations were conducted with projector-augmented wave (PAW) method by Vienna Ab initio Simulation Package (VASP). The exchange-correlation interaction was treated by the generalization gradient approximation (GGA) of Perdew-Burke-Emzerhof (PBE) functional. The energy cutoff for the plane wave basis expansion 450 eV and Gamma centered 3×3×1 k-points mesh were applied to computational process. A $p(2\times4)$ supercell of zinc metal (101) containing two layers was constructed to model zinc anode. The residual force on each atom less than 0.05 eV $Å^{-1}$ was set for convergence criterion of geometry relaxation. The vacuum thickness of the slabs was set to 10 Å to avoid interactions between repeating slabs. To evaluate the Zn affinity on the surface of anode, the adsorption energy was calculated as follows:$E_a = E_{Total} - E_{Zn} - E_{Surface}$, where $E_{Surface}$ and E_{Total} are the total energy of anode before and after Zn adsorption, respectively, while E_{Zn} is the energy of a single Zn atom. The lower the adsorption energy, the stronger the bond between Zn and electrode.

Figures and captions

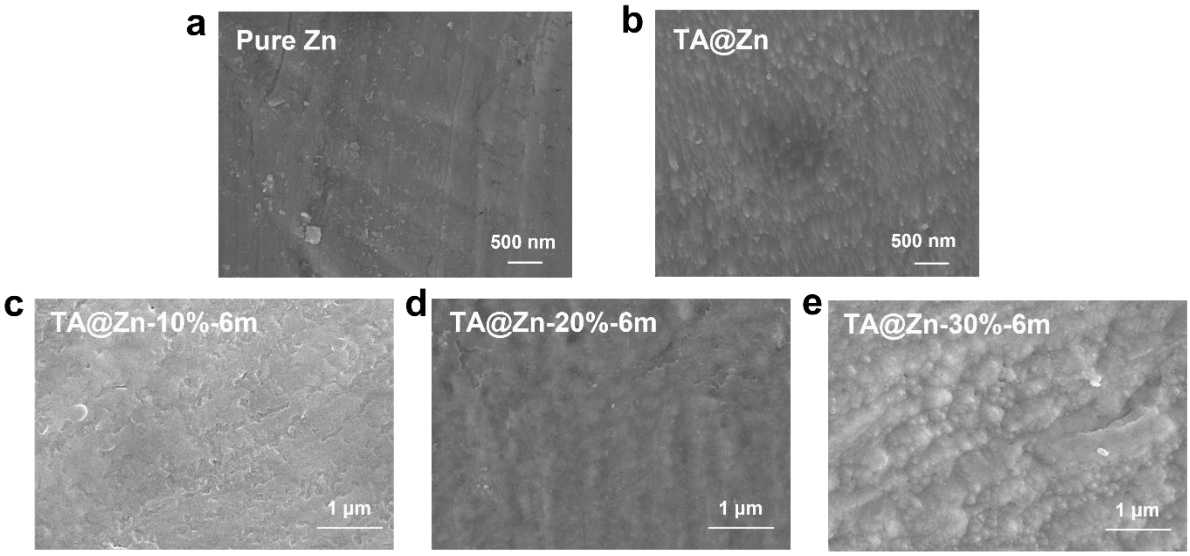

Figure S1 SEM images of (a) pure Zn, (b) TA@Zn, (c) TA@Zn-10%-6 m-, (d) TA@Zn-20%-6m and (e) TA@Zn-30%-6m.

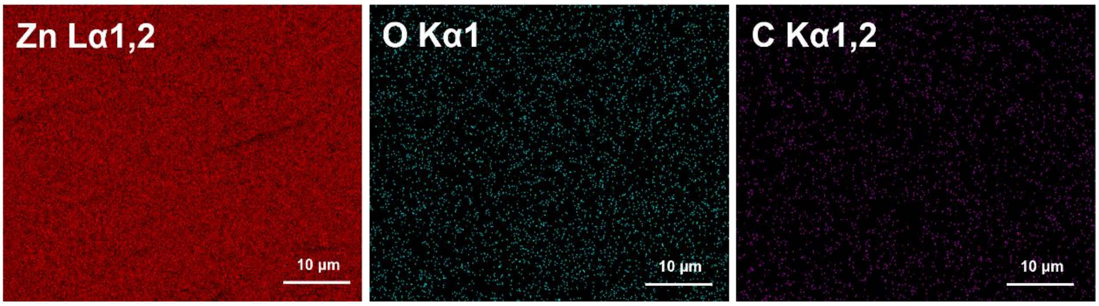

Figure S2 EDS images of TA@Zn.

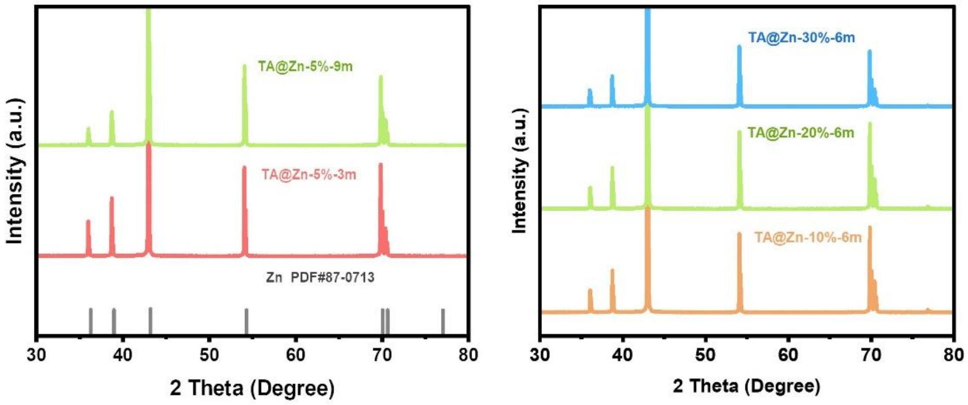

Figure S3 XRD pattern of TA@Zn under different etching conditions.

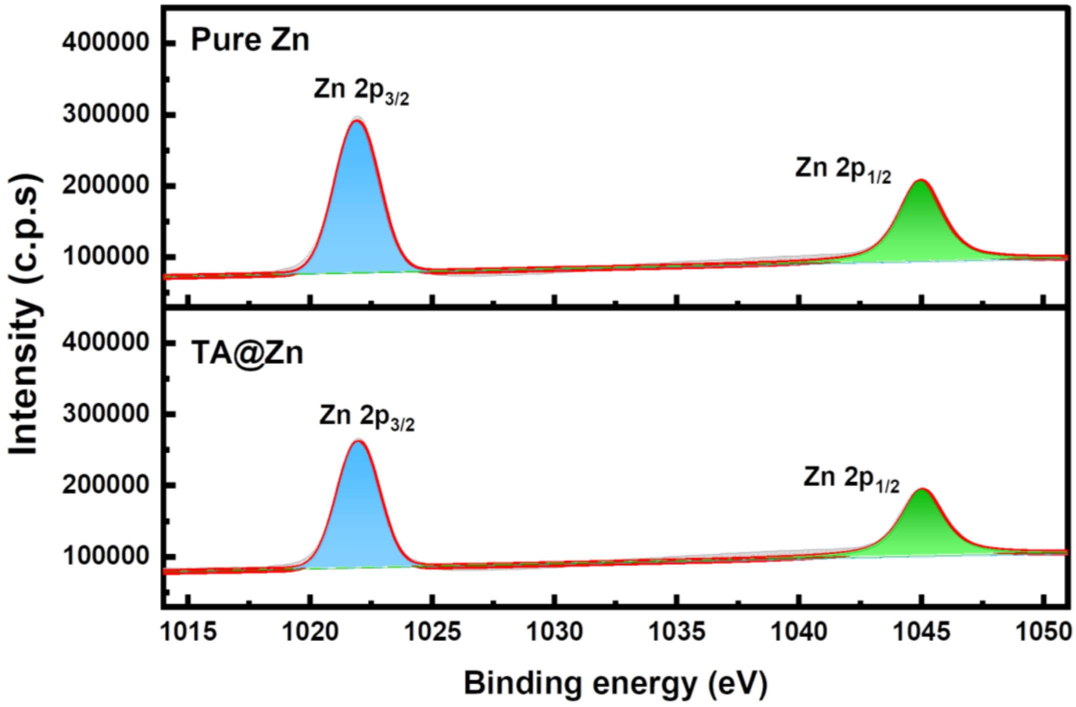

Figure S4 XPS Zn 2p of Zn anode.

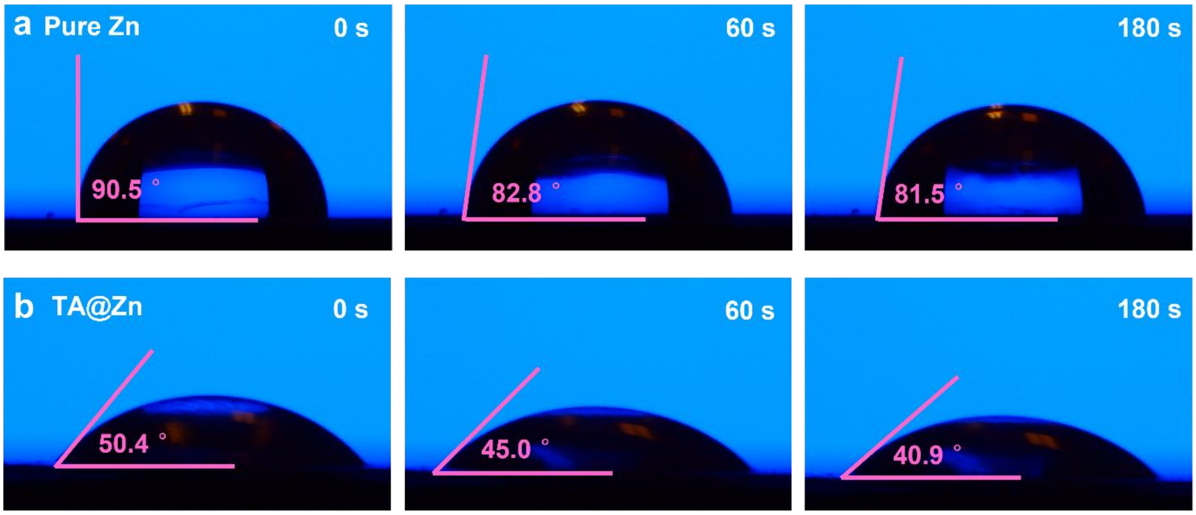

Figure S5 Contact angle measurements of (a) bare Zn and (b) TA@Zn in 0s, 60 s and 180 s of 2 M $ZnSO_4$ aqueous electrolyte.

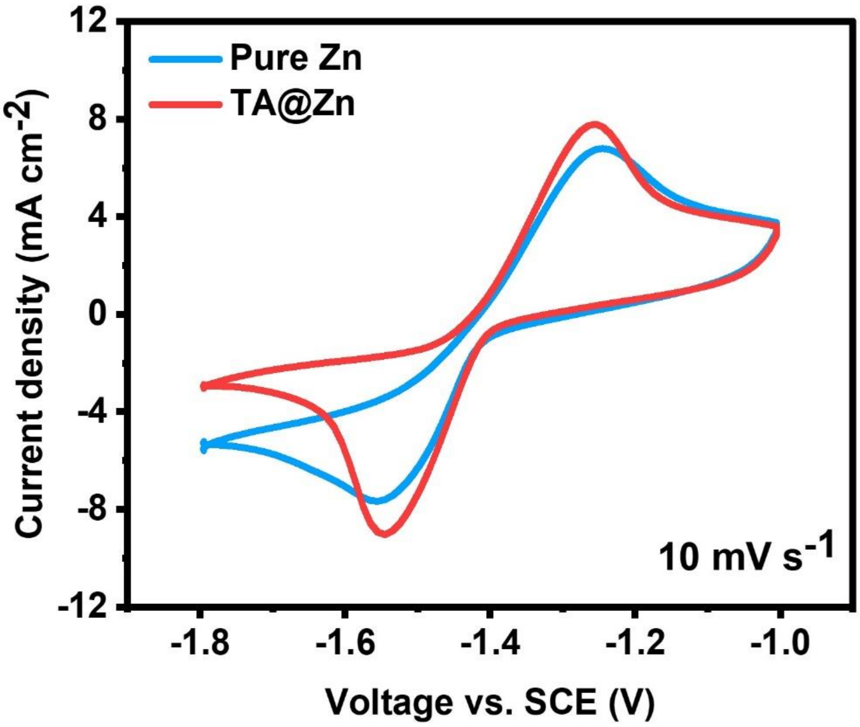

Figure S6 CV profiles of pure Zn and TA@Zn in 0.1 M KOH electrolyte at the scan rate of 10 mV s^{-1}.

.

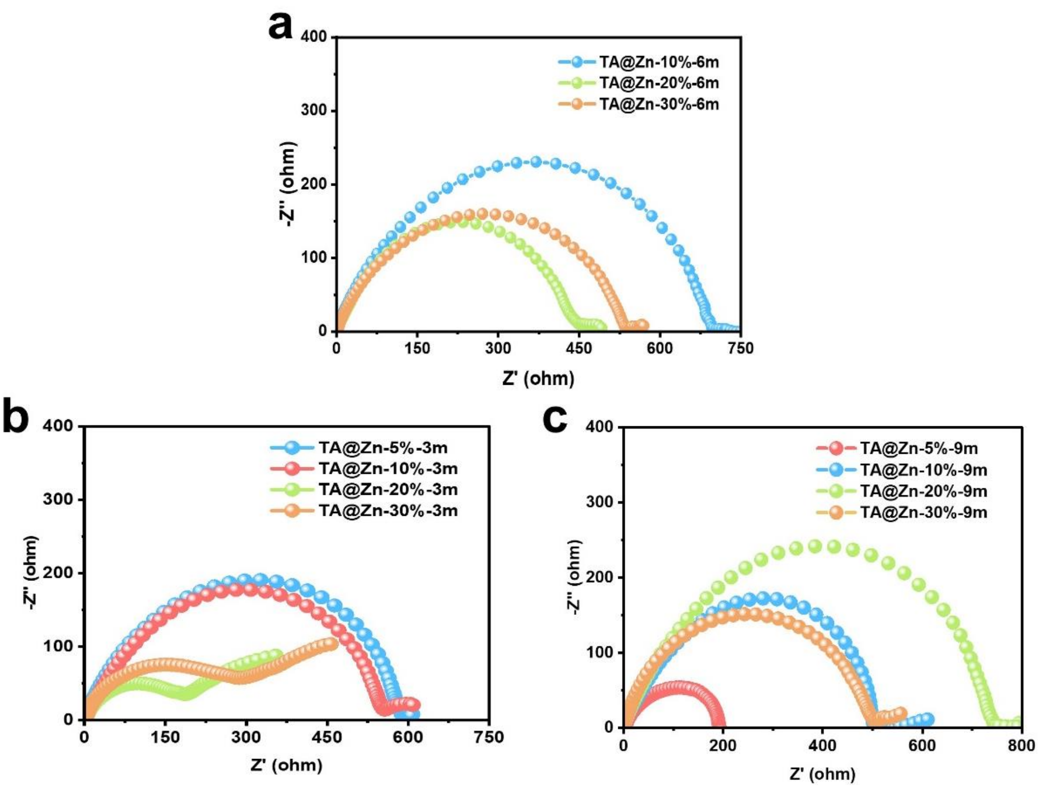

Figure S7 Electrochemical impedances of pure Zn and TA@Zn under etching time of (a) 6m, (b) 3m and (c) 9m.

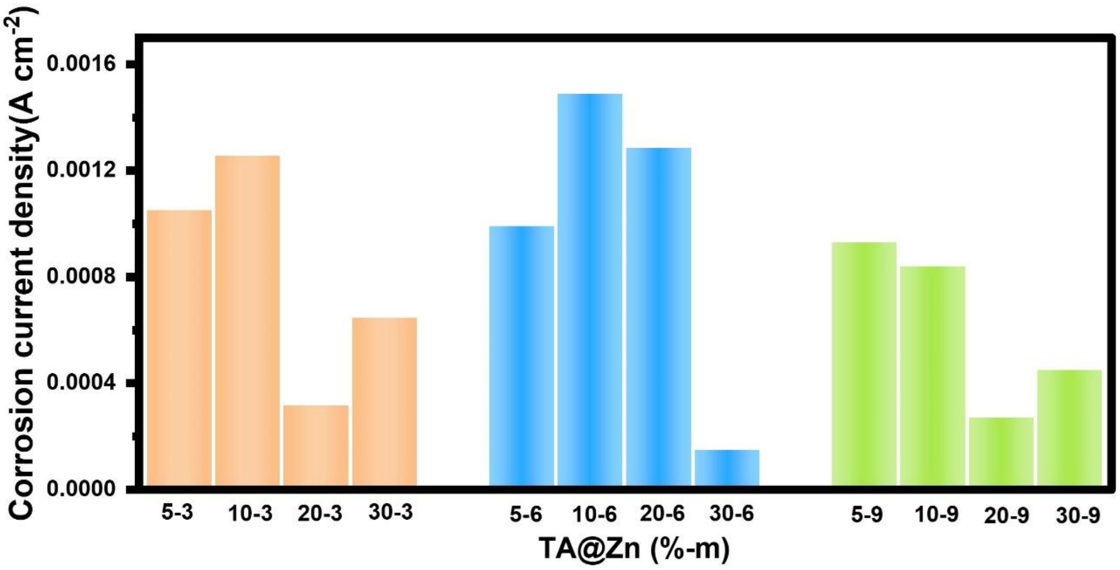

Figure S8 Linear polarization curves of TA@Zn with differen etching condition (e.g., TA@Zn-5%-3m is abbreviated as 5-3).

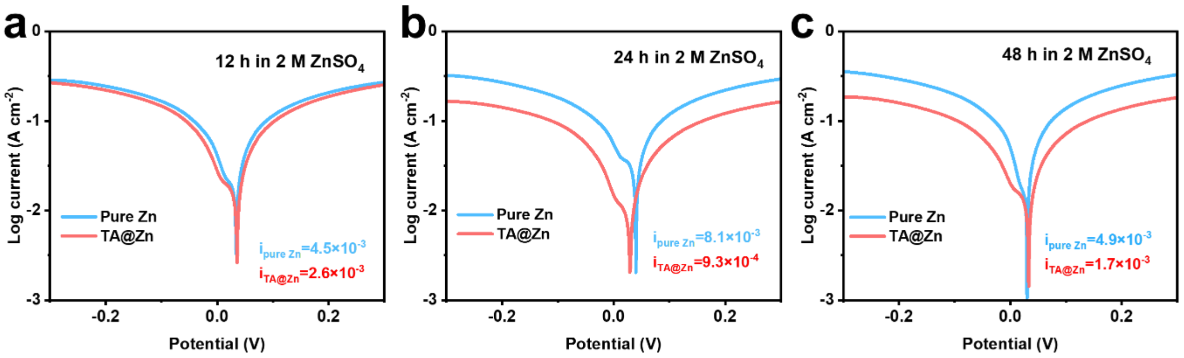

Figure S9 Linear polarization curves of pure Zn and TA@Zn after immersion in 2 M $ZnSO_4$ for (a) 12 h, (b) 24 h and (c) 48 h.

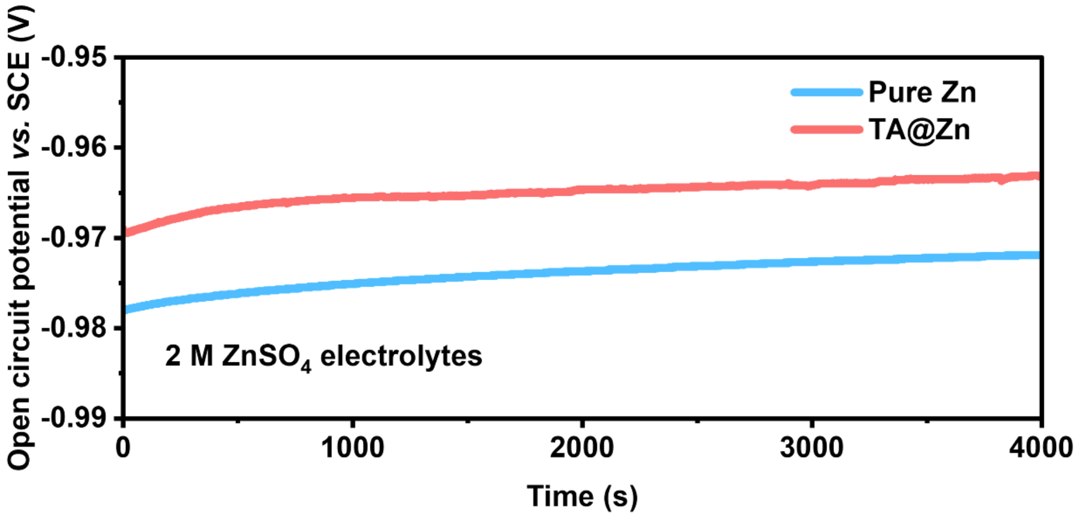

Figure S10 The time-dependent open-circuit voltage spectra of pure Zn and TA@Zn in 2 M $ZnSO_4$ in the range of 4000 s.

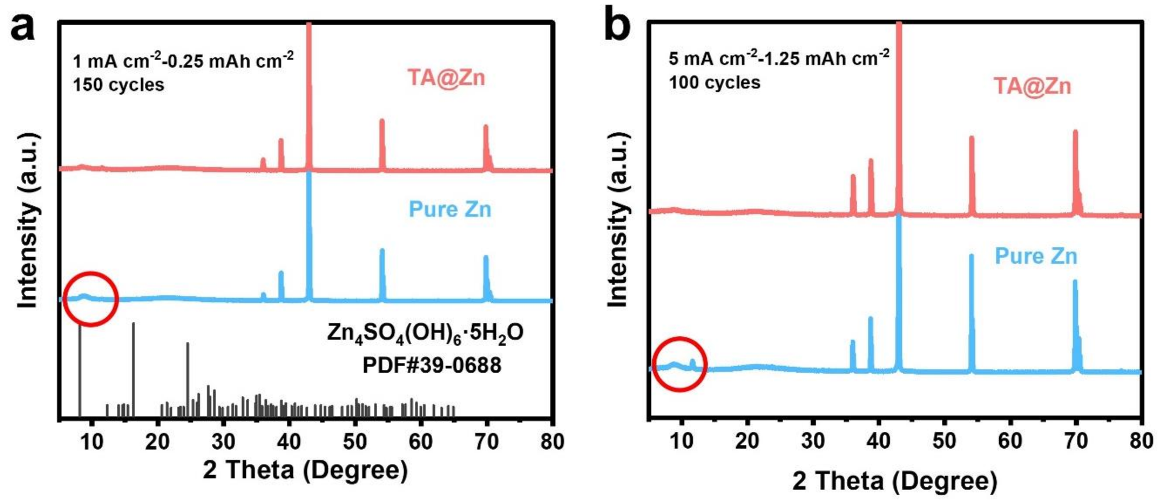

Figure S11 XRD patterns of pure Zn and TA@Zn (a) after 150 cycles at 1 mA cm^{-2}-0.25 mAh cm^{-2} and (b) after 100 cycles at 5 mA cm^{-2}-1.25 mAh cm^{-2}.

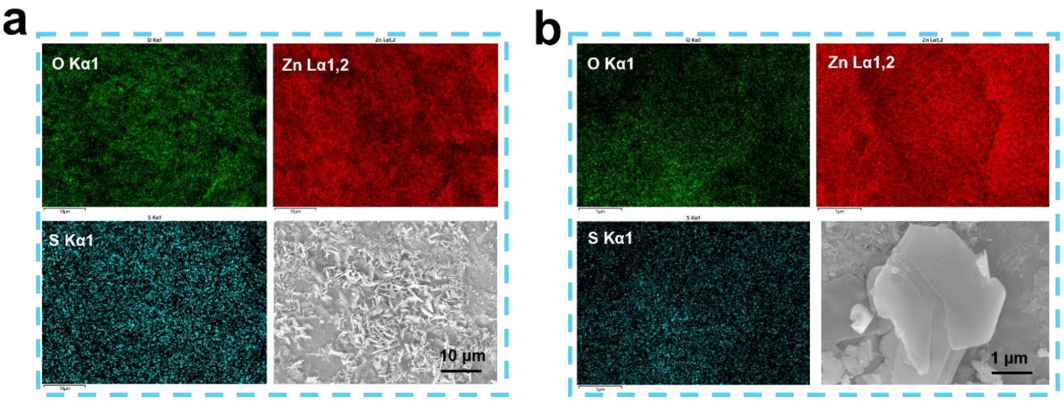

Figure S12 EDS spectra of the surface of TA@Zn after 150 cycles at 1 mA cm^{-2}-0.25 mAh cm^{-2} at (a) 10 μm and (b) 1 μm.

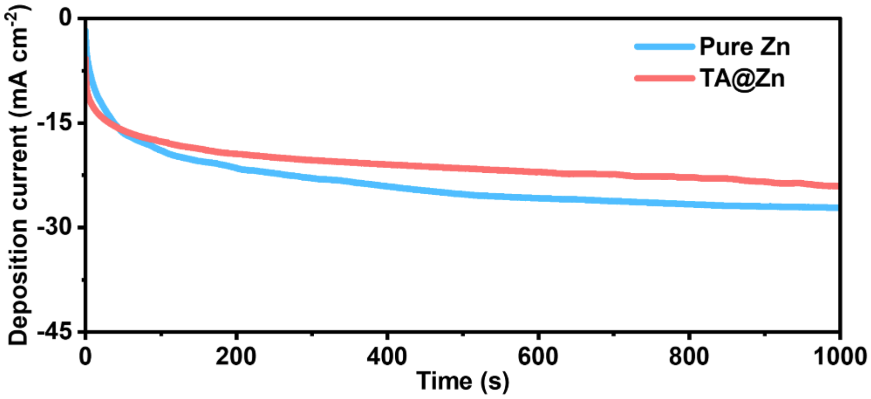

Figure S13 Chronoamperometry curves of Zn|Zn and TA@Zn|TA@Zn symmetric cells.

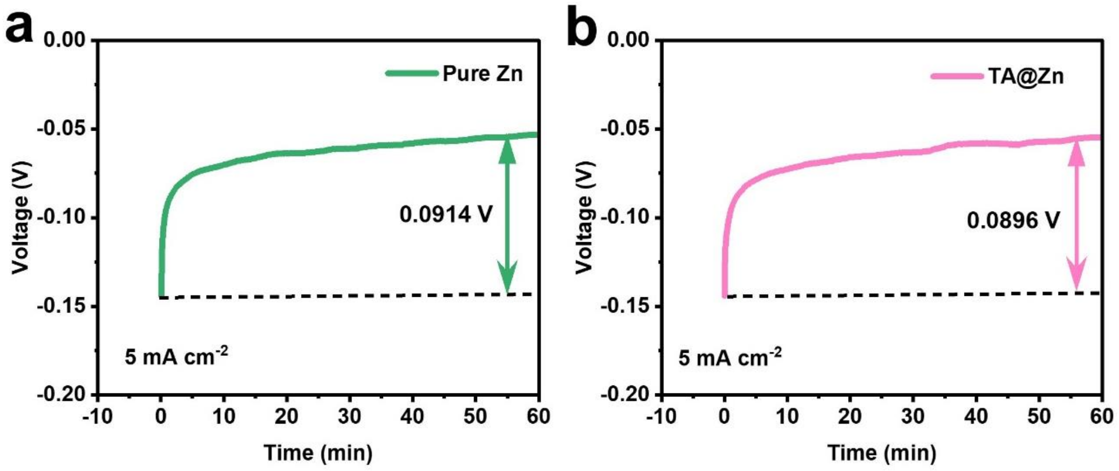

Figure S14 Nucleation overpotential of (a) pure Zn and (b) TA@Zn at 5 mA cm^{-2}.

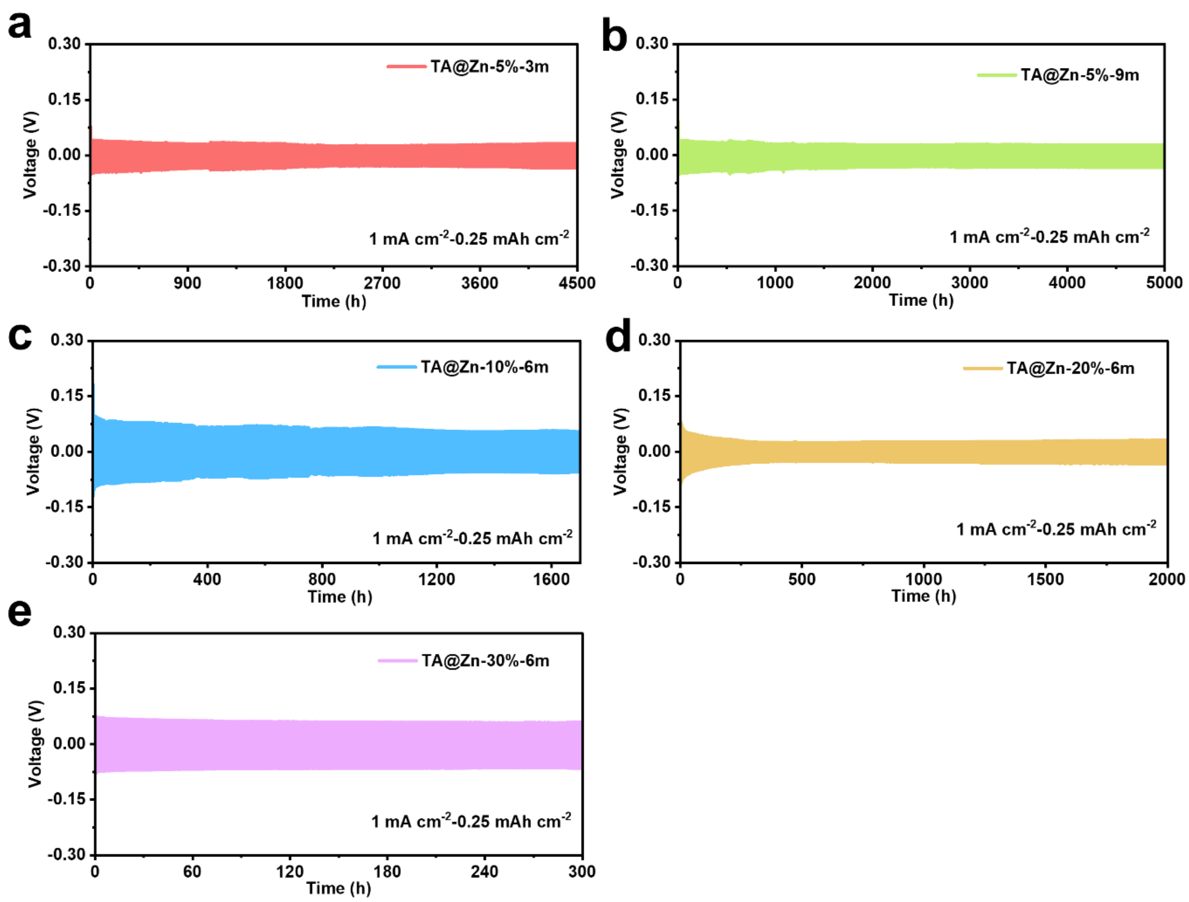

Figure S15 The galvanostatic long-cycle performance of (a) TA@Zn-5%-3m, (b) TA@Zn-5%-9m, (c) TA@Zn-10%-6m, (d) TA@Zn-20%-6m and (e) TA@Zn-30%-6m at 1 mA cm^{-2}-0.25 mAh cm^{-2}.

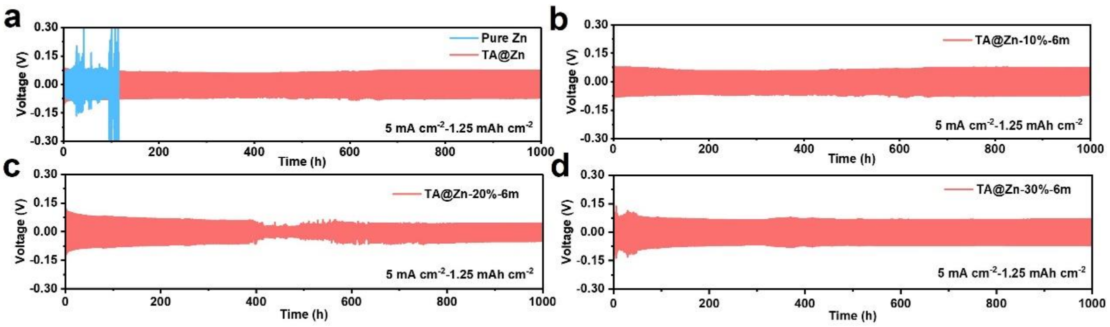

Figure S16 The galvanostatic long-cycle performance of (a) pure Zn and TA@Zn, (b) TA@Zn-10%-6m, (c) TA@Zn-20%-6m and (d) TA@Zn-30%-6m at 5 mA cm^{-2}-1.25 mAh

cm^{-2}.

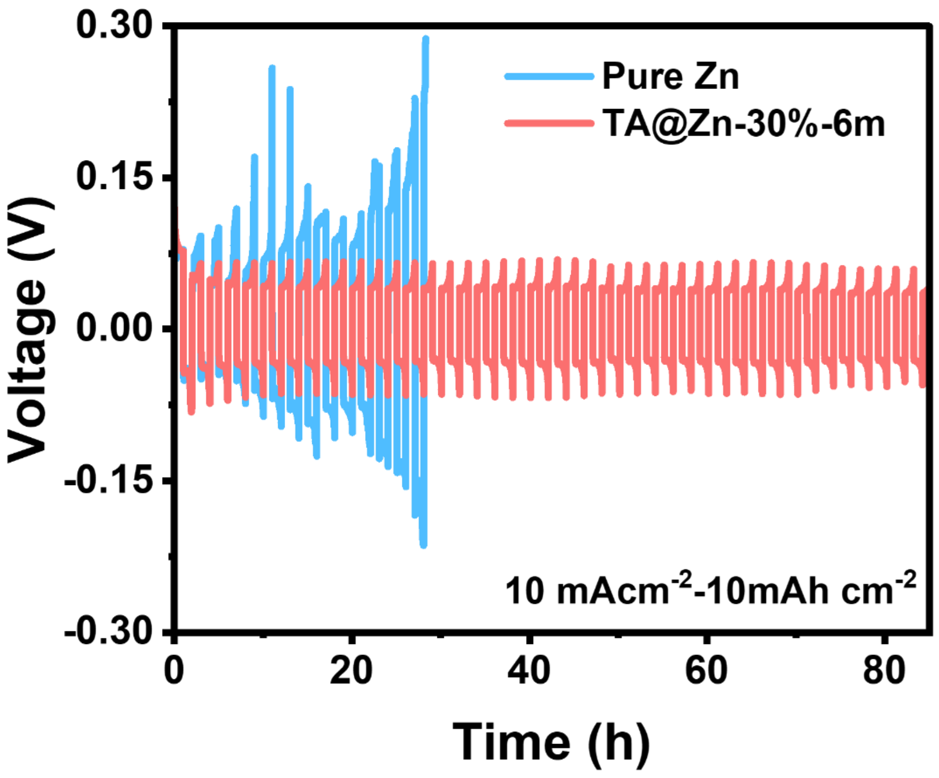

Figure S17 The galvanostatic long-cycle performance of pure Zn and TA@Zn-30%-6m at 10 mA cm^{-2}-10 mAh cm^{-2}.

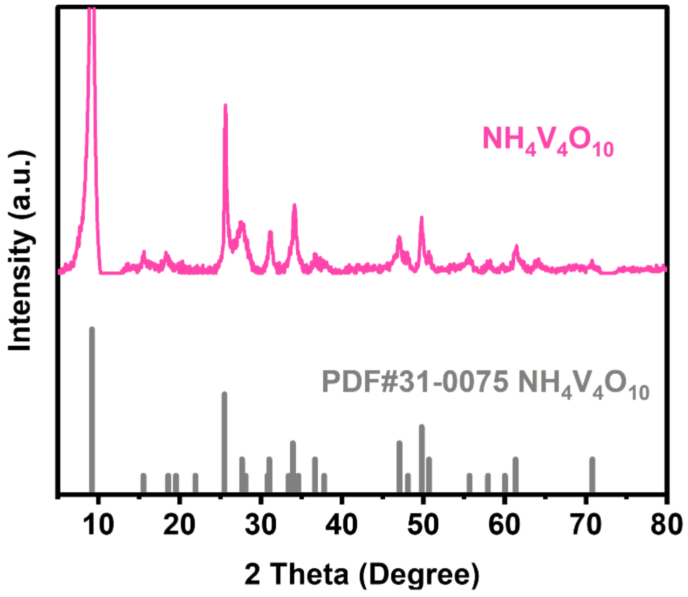

Figure S18 XRD pattern of $NH_4V_4O_{10}$ (NVO).

Table S1 **C 1s, O 1s, and Zn 2p XPS peak assignments for pure Zn and TA@Zn.**

Pure Zn		
	Binding energy (eV)	Chemistry
C 1s	284.29	C-C
	284.99	C-H
	288.36	O=C-O
O 1s	530.63	O=C-O
	531.93	O-H

TA@Zn		
	Binding energy (eV)	Chemistry
C 1s	284.09	C-C
	284.98	C-H
	286.46	C-O-H
	288.66	O=C-O
O 1s	530.68	O=C-O
	532.01	O-H
	532.48	C-O-H

Table S2 Summary of galvanostatic long-cycle performances of pure Zn and the prepared TA@Zn anodes.

	**1 - 0.25**	**5 - 1.25**	**10 – 10**	**20 – 20**
			Unit: mA cm^{-2} – mAh cm^{-2}	
**Pure Zn**	500 h	90 h	Fail	Fail
**TA@Zn**	5000 h	1000 h	100 h	130 h
**TA@Zn-10%-6m**	1700 h	1000 h		
**TA@Zn-20%-6m**	2000 h	1000 h		
**TA@Zn-30%-6m**	300 h	1000 h	85 h	
